# Antipsychotic Polypharmacy among Children and Young Adults in Office-Based or Hospital Outpatient Department Settings

**DOI:** 10.3390/pharmacy5040064

**Published:** 2017-11-23

**Authors:** Minji Sohn, Meghan Burgess, Mohamed Bazzi

**Affiliations:** 1 College of Pharmacy, Ferris State University, 220 Ferris Drive, Big Rapids, MI 49307, USA; bazzim5@ferris.edu; 2 College of Health Professions, Ferris State University, 200 Ferris Drive, Big Rapids, MI 49307, USA; burgem10@ferris.edu

**Keywords:** antipsychotics, polypharmacy, children, adolescents, young adults

## Abstract

The purpose of the study was three-fold: (1) to estimate the national trends in antipsychotic (AP) polypharmacy among 6- to 24-year-old patients in the U.S.; (2) to identify frequently used AP agents and mental disorder diagnoses related to AP polypharmacy; and (3) to assess the strength of association between AP polypharmacy and patient/provider characteristics. We used publicly available ambulatory health care datasets to evaluate AP polypharmacy in office-based or hospital outpatient department settings to conduct a cross-sectional study. First, national visit rates between 2007 and 2011 were estimated using sampling weights. Second, common diagnoses and drugs used in AP polypharmacy were identified. Third, a multivariate logistic regression model was developed to assess the strength of association between AP polypharmacy and patient and provider characteristics. Between 2007 and 2011, approximately 2% of office-based or hospital outpatient department visits made by 6- to 24-year-old patients included one or more AP prescriptions. Of these visits, 5% were classified as AP polypharmacy. The most common combination of AP polypharmacy was to use two or more second-generation APs. Also, bipolar disorder and schizophrenia were the two most frequent primary mental disorder diagnoses among AP polypharmacy visits. The factors associated with AP polypharmacy were: older age (young adults), black, having one or more non-AP prescriptions, and having schizophrenia or ADHD.

## 1. Introduction

Over the last two and half decades, a wide variety of psychotropic medications have reinvented psychiatric therapy, especially for children and young adults. In particular, the profession of medicine has observed an increased frequency in the use of antipsychotic (AP) medication in the age group [1,2,3,4]. First-generation, typical APs were developed in the 1950s, and second-generation APs (i.e., also known as atypical APs) were developed in the 1990s. Second-generation APs boasted reduced extrapyramidal symptoms and other health problems caused by the use of first-generation APs [5,6]. As a result, the overall utilization of APs has increased significantly over time in patients of all ages, including children [7,8,9,10,11,12,13,14,15,16]. According to a study by Sieda et al., by 1996, APs were prescribed for 8.6 per 1000 children. By 2002, this statistic rose drastically to include as many as 39.4 per 1000 children [17].

In the midst of the increasing use of APs in children, AP polypharmacy is a particular area of concern. In some cases, using two or more medications to treat a mental illness may be more effective than monotherapy, especially if the mechanisms of action are complementary [18,19]. However, the concomitant use of two or more pharmacologically similar APs lacks scientific rationale and evidence. One may argue that using two similar agents may result in additive efficacy or rapid therapeutic response, but this argument is not supported by any compelling evidence or theoretical explanation [20,21]. Instead, AP polypharmacy increases the risk of drug overdosing and adverse side effects, such as hyperlipidemia or type 2 diabetes [20,21,22,23].

Several risk factors of AP polypharmacy have been discussed elsewhere. Non-elderly patients (age < 65) [24,25], patients in an inpatient setting [26,27], particularly those with a longer stay [26,28], and schizophrenia [29,30,31] are reported as being associated with AP polypharmacy. The impact of patient sex on AP polypharmacy is inconclusive [24,27,32]. However, a majority of these studies primarily focus on adult populations, and AP polypharmacy trends in children are underexplored.

The purpose of this study was to assess the U.S. national trends in AP polypharmacy among children and young adults in office-based or hospital outpatient department settings. More specifically, we carried out three specific objectives: (1) evaluate the yearly trends in AP polypharmacy use amongst 6- to 24-year-old patients in the U.S.; (2) identify frequently used AP agents and the primary mental diagnoses in AP polypharmacy; and (3) assess the strength of association between AP polypharmacy and the characteristics of patients and providers.

## 2. Methods

### 2.1. Data Source

The cross-sectional dataset for the study was obtained from the National Ambulatory Medical Care Survey (NAMCS) and the National Hospital Ambulatory Medical Care Survey (NHAMCS) database. The NAMCS and NHAMCS are nationally representative surveys that collect samples on patient visits to office-based or hospital outpatient department-based providers who are primarily engaged in direct patient care. More specifically, the sampling process utilizes a three-stage probability design. The first stage of probability sampling is based on geographic segments, and the second stage involves the probability sampling of physician practices. Thirdly, within the sampled physician practices, random samples of patient visits are collected. Each NAMCS/NHAMCS record has a single sample weight that is calculated based on this three-stage probability design. The NAMCS/NHAMCS data contains patient demographics, physician specialty, other clinicians seen during the visits, diagnoses based on the International Classification of Diseases, Ninth Revision, Clinical Modification (ICD-9-CM), and up to eight prescribed drugs during the visit. Our study sample consisted of AP visits (refer to the succeeding text for details on defining AP visits) of patients who were 6 to 24 years of age between 2007 and 2011. We intended to estimate the national trends in non-emergent visits associated with AP prescriptions, and therefore, we excluded data from the hospital emergency department from the study. The rationale is that treatment strategies can be different between an emergency department setting and an office-based or hospital outpatient department setting. For example, pharmacotherapy in an emergency department setting is likely to be focused on treating a particular incident or episode in the short-term, rather than for the chronic management of mental illness with regular follow-up visits. Sample weights were applied in all analyses using Stata Version 13 (StataCorp, College Station, TX, USA, 2013). The data use for the study was approved by the Ferris State University Institutional Review Board, which oversees the ethical conduct of research at this institution.

### 2.2. National Trends in AP Polypharmacy

As the first objective of the study, we estimated the national AP visit rates and the national AP polypharmacy visit rates in each year between 2007 and 2011. Since the unit of observation of the dataset is the physician-patient encounter, the number of patients cannot be estimated. A patient visit was defined as an AP visit if one or more of the following medications were prescribed: (1) as typical APs, haloperidol, loxapine, thiothixene, trifluoperazine, chlorpromazine, fluphenazine, perphenazine, prochlorperazine, and thioridazine; and (2) as atypical APs, risperidone, olanzapine, quetiapine, ziprasidone, aripiprazole, paliperidone, asenapine, iloperidone, and clozapine. An AP polypharmacy visit was defined as an AP visit with two or more AP prescriptions. Drug prescriptions were identified using the Multum classification system (Cerner Corporation, Lexicon, Denver, CO, USA). The Multum classification utilizes a three-level nested category system in which drugs are coded in terms of their generic components and therapeutic classes.

### 2.3. Diagnoses and Frequently Used APs in AP Polypharmacy

As the second objective of the study, we identified mental disorder diagnoses (ICD-9-CM code 290–319) and frequently used AP agents associated with AP polypharmacy. If two or more mental disorder diagnoses were present, we identified a primary mental disorder diagnosis based on a previously developed hierarchy [1]. More specifically, the primary mental disorder diagnosis was assigned in the following order: (1) schizophrenia and other psychoses (295, 297–298); (2) pervasive developmental disorder or mental retardation (299, 317–319); (3) bipolar disorder (290.6, 296.1, 296.4–296.8, 301.13); (4) disruptive behavior disorder (312.0–312.4, 312.81, 312.82, 312.89, 312.9, 313.81); (5) attention-deficit/hyperactivity disorder (ADHD) (314); (6) depression/mood disorder, not otherwise specified (293.83, 296.2, 296.3, 296.9, 298.0, 300.4, 311); (7) anxiety disorder (293.84, 300.0, 300.2, 300.3, 309.3, 309.21, 309.81, 313.0, 313.2, 313.89); (8) adjustment disorder (308.0–308.2, 308.4, 308.9, 309.0–309.4, 309.82, 309.83, 309.89, 309.9); (9) communication and learning disorder (307.0, 307.9, 315.0–315.2, 315.31, 315.32, 315.39, 315.9); and (10) other mental disorders (290–319, not listed above). The assignment of primary mental disorder was mutually exclusive. We adopted this method because the ordering of the diagnostic group generally corresponds to the strength of clinical evidence for AP treatment in pediatrics [1,11].

### 2.4. Patient and Provider Characteristics Associated with AP Polypharmacy

We assessed the association between patient and provider characteristics and AP polypharmacy. As patient characteristics, age, sex, race, geographic region (Northeast, Midwest, South, and West), Metropolitan Statistical Area (MSA), primary payer source, median household income based on patient zip code, and % of adults with a Bachelor’s degree or higher based on patient zip code were included in the model. As provider characteristics, we identified variables on health care providers (e.g., psychiatrist, mental health provider) and the type of health care services provided during the visit (e.g., psychotherapy, other mental health counseling). A mental health provider refers to psychologists, counselors, social workers, or therapists who provide mental health counseling.

Differences in patient and provider characteristics between monotherapy and polypharmacy visits were tested for statistical significance. We used chi-squared tests for categorical variables and *t*-tests for continuous variables. Also, univariate and multivariate logistic regression models were developed to estimate the strength of association between polypharmacy and patient and provider characteristics. 

In the NAMCS/NHAMCS, between 2007 and 2011, approximately 18% of all patient visits did not have race information. For the observations missing race information, we used imputed values that were provided by the NAMCS/NHAMCS. The imputation methods used by the NAMCSNHAMCS are described in the Public Use Data File Documentation [33]. For variables of median household income based on patient zip code and % of adults with a Bachelor’s degree or higher based on patient zip code, approximately 15% of all patient visits had missing data. Since the NAMCS/NHAMCS does not provide the patient zip code or imputation values for these variables, we created a missing data indicator for the variables and treated them as a separate category. Then, we conducted a sensitivity analysis by excluding missing values from the estimation to check whether the findings were robust.

## 3. Results

### 3.1. National Rates of AP Polypharmacy

**Figure 1:**
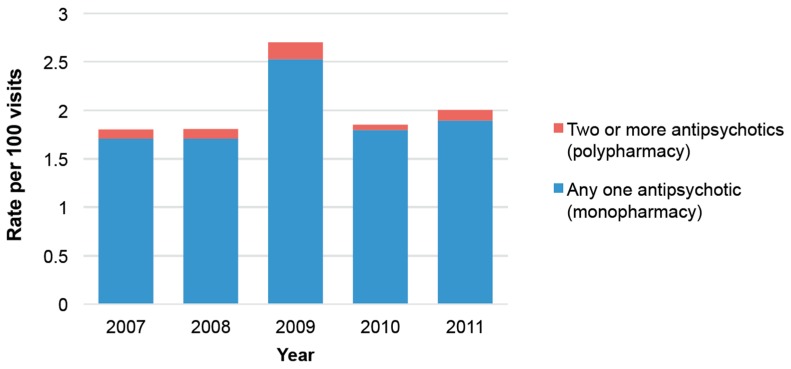
National antipsychotic (AP) visit rates and the proportion of AP polypharmacy between 2007 and 2011.

Between 2007 and 2011, approximately 1.92 per 100 visits included one or more AP prescriptions (95% Confidence interval (95% CI) 1.65–2.25 per 100 visits, weighted count 16,131,721). Of these visits, 5% were visits related to AP polypharmacy (95% CI 3.77–7.78 weighted count 877,071). Year-by-year estimation is shown in Figure 1. Over the 5-year observation period, trends in AP visits and AP polypharmacy did not increase significantly. Although not significant, the AP visit rate appeared higher in 2009, as it was 2.53 per 100 visits (95% CI 1.93–3.31 per 100 visits), while it was between 1.71 and 1.90 per 100 visits in other years (i.e., 1.71; 95% CI 1.24–2.35 per 100 visits in 2008, 1.90; 95% CI 1.41–2.55 per 100 visits in 2011). As for AP polypharmacy, year-to-year variation ranged from 3% (95% CI 1.84–4.47 in 2010) to 7% (95% CI 3.12–14.76 in 2009).

### 3.2. Common Mental Disorder Diagnoses and Drugs Used in AP Polypharmacy

**Figure 2:**
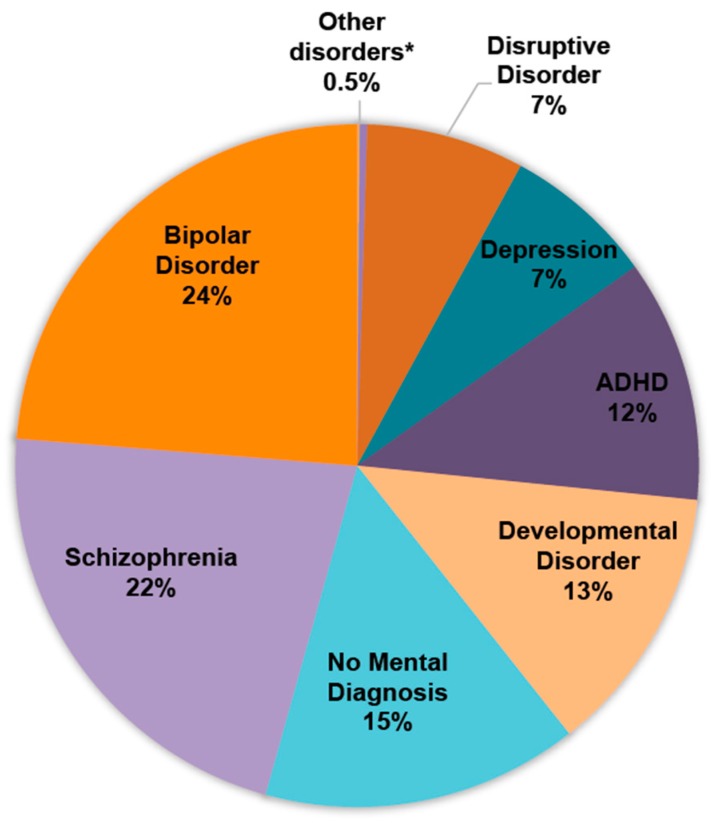
Primary mental disorder diagnoses in AP polypharmacy. Percentages may not total 100% due to rounding. * Anxiety disorder, adjustment disorder, communication and learning disorder, and other mental disorders.

Bipolar disorder and schizophrenia were the most common primary mental diagnoses in AP polypharmacy (24% and 22%, respectively) (Figure 2). Approximately 12% were patients diagnosed with ADHD, and 15% did not have any recorded mental disorder diagnosis.

In AP polypharmacy visits, the most common combination was to use two or more second-generation APs (80.63%, 95% CI 60.20–91.97). The combination of first- and second-generation APs accounted for 19.37% (95% CI 8.03–39.80) (Table 1). Among the drugs used in AP polypharmacy, quetiapine was the most frequently used agent (53.25%; 95% CI 37.45–68.41), followed by aripiprazole (48.46%; 95% CI 33.58–63.62) (Table 2).

### 3.3. Factors Associated with AP Polypharmacy

Patient and provider characteristics were compared between AP monotherapy and AP polypharmacy visits (Table 3). Among those, the variables of patient age, whether mental health provider was seen (yes/no), and the number of non-AP prescribed were statistically significant. More specifically, a higher proportion of AP polypharmacy visits was made by young adult patients (age 19–24), and a smaller proportion was made by elementary school-aged children (age 6–12), compared to AP monotherapy visits (*p* = 0.004). Also, a higher proportion of AP polypharmacy visits involved a mental health provider during the visit than AP monotherapy visits. (20.57% versus 8.06%, *p* = 002). The average number of non-AP prescriptions was 2.45 in AP polypharmacy visits, while it was 2.02 in AP monotherapy visits (*p* = 0.03).

In the multivariate logistic regression model, the variables of age, race, number of non-AP prescriptions, schizophrenia, and ADHD were significantly associated with AP polypharmacy (Table 4). Young adults were more likely to have AP polypharmacy visits than elementary school-aged children (adjusted odds ratio (AOR) 3.43; 95% CI 1.07–11.02). Adolescents were not significantly different from elementary school-aged children in terms of the rate of AP polypharmacy (AOR 1.65; 95% CI 0.56–4.89). With respect to race, compared to white patients, black patients were significantly less likely to have AP polypharmacy (AOR 0.21; 95% CI 0.07–0.57). Also, compared to AP visits without any concomitant non-AP prescriptions, AP visits with one non-AP prescription were more likely to have AP polypharmacy (AOR 5.57; 95% CI 1.65–18.86). AP visits with two or more non-AP prescriptions showed a similar association with AP polypharmacy (two non-AP AOR 8.08; 95% CI 2.01-32.48, three or more non-AP AOR 6.67; 95% CI 2.07–21.53). As for primary mental disorder diagnosis, compared to AP visits with a bipolar disorder diagnosis, AP visits with a schizophrenia or an ADHD diagnosis were more likely to have AP polypharmacy (AOR 4.23; 95% CI 1.61–11.16, AOR 2.65; 95% CI 1.07–6.60, respectively). The variables of sex, geographic region, MSA, payer source, psychotherapy, other mental health counseling, health care provider, household income, and education level were not significantly associated with AP polypharmacy.

As a sensitivity analysis, we excluded observations missing either median household income based on patient zip code or % of adults with a Bachelor’s degree or higher based on patient zip code from the estimation. As a result, the association between AP polypharmacy and these variables remained as not significant (*p* >0.05) in the chi-squared tests and logistic regressions (Appendix A).

## 4. Discussion

The purpose of the study was: (1) to estimate the national trends in AP polypharmacy among 6- to 24-year-old patients in the U.S.; (2) to identify frequently used AP agents and diagnoses related to AP polypharmacy; and (3) to assess the strength of association between AP polypharmacy and patient/provider characteristics.

Although not significant, the rate of AP visits and the proportion of AP polypharmacy appeared increased in 2009 and decreased in 2010. Several factors may have affected this trend. For example, in 2009, two second-generation AP agents, iloperidone and asenapine, were newly approved by the U.S. Food and Drug Administration (FDA), and aripiprazole was additionally approved for the treatment of autistic spectrum disorder. New approvals of APs and drug indications could have temporarily increased the rate of AP prescription and polypharmacy in 2009. In addition, new findings of clinical trials and observational studies associated with APs, changes in practice guidelines, pharmaceutical marketing, and public education about the effectiveness and risks of APs would potentially have increased or decreased the rate of AP prescription to a certain extent.

Bipolar disorder and schizophrenia were the two most prevalent primary mental diagnoses among AP polypharmacy visits (24% and 22%, respectively). Approximately 12% of AP polypharmacy visits had ADHD. However, unlike bipolar disorder or schizophrenia, none of the APs are approved for the treatment of ADHD by the FDA. While the off-label use of APs in ADHD patients has been reported in a number of studies, our study further extends the concern to AP polypharmacy. It is concerning that a risk of unnecessary harm from AP misuse is imposed on ADHD patients, not only from the off-label use perspective but also from the polypharmacy perspective.

Approximately 15% of AP polypharmacy visits did not have any mental disorder diagnosis. It should be noted that the NAMCS/NHAMCS collects only up to three diagnosis codes, and therefore, if a patient has three or more physical disorder diagnoses, it is possible that a mental disorder diagnosis is not captured in the dataset due to limited space in the survey. However, in our study sample, of the AP polypharmacy visits without any mental disorder diagnosis, 83% had two or fewer diagnoses. This means that approximately 12% of AP polypharmacy visits did not have any mental disorder diagnosis, and limited space in the survey was not the reason for it.

The most common combination of AP polypharmacy was to use two or more second-generation APs (80.63%). This trend can be explained by that second-generation APs have no or reduced extrapyramidal symptoms compared to the first-generation APs. When second-generation APs were introduced, they were marketed as relatively safer agents than the pre-existing first-generation APs [5,6]. However, great caution is needed before using second-generation APs, since they have serious side effects, including hyperlipidemia and type 2 diabetes [20,21,22,23]. Furthermore, the concomitant use of two or more second-generation antipsychotics may only increase the risk of those adverse events.

Among AP visits, polypharmacy visits were significantly associated with young adults than elementary school-aged children, and black patients than white patients. Also, patients who had one or more non-AP prescriptions in addition to their AP prescription were significantly more likely to be classified as AP polypharmacy. Interestingly, when adjusted for other covariates, primary payer source, household income, and education-related factors were not significantly associated with AP polypharmacy. In previous studies, the initiation of AP treatment in young patients was significantly associated with socioeconomic factors, such as having Medicaid as the primary payer source [3,4,23]. These findings suggest that, when it comes to AP polypharmacy, individuals’ comorbidities and the complexity of the physical/mental conditions play a more important role than previously reported risk factors.

Some limitations should be noted. First, the unweighted count of AP polypharmacy in each year was small (*n* < 30), and therefore, the yearly estimation of AP polypharmacy can be potentially unreliable. In order to address the small sample size problem, we combined five-years’ worth of data (2007–2011) and used it to carry out the objectives of the study. Nonetheless, caution is needed when interpreting the results of the study, particularly for the yearly estimation (Figure 1). Second, the NAMCS/NHAMCS collects the cross-sectional sample of office-based or hospital outpatient department visits and it does not establish the sequence of events. For example, our analysis cannot infer that the concurrent prescription of non-AP drugs caused the AP polypharmacy. Instead, we only suggest that the number of non-AP prescriptions is positively associated with the likelihood of AP polypharmacy. In addition, due to the cross-sectional nature of the study dataset, our study cannot exclude the short-term, temporary use of two or more APs from the definition of AP polypharmacy. This could overestimate the true rate of AP polypharmacy because it is common to have a short overlap period when a patient switches APs. Third, our study sample only includes non-emergent office-based or hospital outpatient department visit data, and it does not include emergency room or inpatient visit data. This would result in the underestimation of AP polypharmacy rates. It is because AP polypharmacy occurs most frequently in schizophrenia or bipolar disorder patients, and patients with these conditions are more frequently hospitalized. Therefore, it should be noted that the findings of the study should not be applied to an emergency room or inpatient visits. Fourth, we conducted a multivariate logistic regression model to adjust for a number of potential confounders, but it is limited to variables that are observable and available in the dataset. While our model includes variables that were identified in prior studies, we cannot rule out the possibility of having a confounding bias.

In conclusion, between 2007 and 2011, approximately 2% of office-based or hospital outpatient department visits made by 6- to 24-year-old patients included one or more AP prescriptions. Of these visits, 5% were AP polypharmacy visits. The most common combination of AP polypharmacy was to use two or more second-generation APs concomitantly. Also, bipolar disorder and schizophrenia were the two most frequent primary mental diagnoses among AP polypharmacy visits. The factors associated with AP polypharmacy were: older age (young adults), black, having one or more non-AP prescriptions, and having schizophrenia or ADHD.

## Figures and Tables

**Table 1:** Frequently used antipsychotic classes in AP polypharmacy. *

Drug Class Used in AP Polypharmacy	%	95% Confidence Interval
Second generation only	80.63	60.20–91.97
First and second generations	19.37	8.03–39.80

* The number of observations for the “first generation only” category was very small (unweighted count = 5), and it was estimated to be less than 0.001% of AP polypharmacy visits.

**Table 2:** Frequently used antipsychotics in AP polypharmacy.

Drugs Used in AP Polypharmacy	%	95% Confidence Interval
First generation antipsychotics		
Haloperidol	12.19	3.08–37.77
Chlorpromazine	6.98	2.24–19.72
Prochlorperzine	0.11	0.02–0.83
Fluphenazine	0.09	0.01–0.65
Second generation antipsychotics		
Quetiapine	53.25	37.45–68.41
Aripiprazole	48.46	33.58–63.62
Olanzapine	26.1	13.60–44.21
Risperidone	26.41	15.18–41.89
Ziprasidone	23.17	9.99–45.04
Clozapine	8.09	2.77–21.40
Paliperidone	7.56	1.13–36.89

**Table 3:** National estimated visit rates of AP monotherapy and AP polypharmacy stratified by patient and provider characteristics between 2007 and 2011.

Characteristics	Monotherapy		Polypharmacy		*p*-Value *
	Weighted Count (in Thousands)	Weighted %	Weighted Count (in Thousands)	Weighted %	
Age					0.004
6–12 (Elementary school age)	4251	27.87	135	15.44	
13–18 Years (Adolescent)	5980	39.2	227	25.91	
19–24 (Young adult)	5023	32.93	514	58.66	
Sex					0.084
Female	5803	38.04	218	24.84	
Male	9452	61.96	659	75.16	
Race					0.365
White	12,255	80.34	760	86.62	
Black	2357	15.45	81	9.18	
Other/Unspecified	642	4.21	37	4.2	
Geographic region					0.685
Northeast	3485	22.85	273	31.1	
Midwest	3004	19.69	168	19.13	
South	5863	38.44	311	35.5	
West	2902	19.03	125	14.27	
MSA or non-MSA area					0.291
MSA	13,378	87.7	807	92.04	
non-MSA	1877	12.3	70	7.96	
Payer					0.152
Private	5666	37.45	276	31.53	
Medicaid	6576	43.46	316	36.03	
Other	2888	19.09	285	32.45	
Psychiatrist					0.317
Yes	8261	54.16	552	62.93	
No	6693	45.84	325	37.07	
Psychotherapy					0.077
Yes	4706	30.85	396	45.2	
No	10,549	69.15	481	54.8	
Other mental health counseling					0.872
Yes	3504	22.97	210	23.99	
No	11,751	77.03	667	76.01	
Mental health provider					0.002
Yes	1230	8.06	180	20.57	
No	14,025	91.94	697	79.43	
Number of non-AP (mean, SD)	2.02	0.08	2.45	0.20	0.03
Median household Income based on patient zip code					0.381
Quartile 1	3302	21.65	225	25.60	
Quartile 2	3294	21.59	78	8.93	
Quartile 3	3100	20.32	184	21.01	
Quartile 4	4271	28.01	331	37.77	
Missing data	1287	8.44	59	6.69	
% of Adults with a Bachelor’s degree or higher based on patient zip code					0.911
Quartile 1	2973	19.49	154	17.52	
Quartile 2	3490	22.88	165	18.81	
Quartile 3	3103	20.34	217	24.73	
Quartile 4	4401	38.85	283	32.24	
Missing data	1287	8.44	59	6.69	

* Chi-squared tests were used for all variables, except the number of non-AP prescriptions (*t*-test). MSA: Metropolitan Statistical Area.

**Table 4:** Univariate and multivariate logistic regressions examining factors associated with AP polypharmacy.

Characteristics	Unadjusted Odds Ratio	95% Confidence Interval	Adjusted Odds Ratio *	95% Confidence Interval
Age				
6–12 (Elementary school age)	1	Reference	1	Reference
13–18 (Adolescent)	0.54	0.28–1.05	1.65	0.56–4.89
19–24 (Young adult)	2.89	1.67–5.01	3.43	1.07–11.02
Sex				
Male	1	Reference	1	Reference
Female	0.54	0.26–1.10	0.51	0.22–1.17
Race				
White	1	Reference	1	Reference
Black	0.55	0.27–1.15	0.21	0.07–0.57
Other/Unspecified	1	0.26–3.82	0.87	0.16–4.79
Payer				
Private	1	Reference	1	Reference
Medicaid	0.82	0.41–1.62	1.27	0.54–3.01
Other	2.06	0.98–4.32	2.27	1.00–5.18
Psychotherapy	1.85	0.93–3.69	1.57	0.69–3.59
Other mental health counseling	1.06	0.53–2.11	0.98	0.45–2.13
Mental health provider	2.95	1.45–6.00	2.24	0.86–5.65
Psychiatrist	1.44	0.71–2.93	1.75	0.78–3.94
Number of non-AP				
None	1	Reference	1	Reference
One	0.91	0.49–1.69	5.57	1.65–18.86
Two	1.42	0.57–3.56	8.08	2.01–32.48
Three or More	1.25	0.63–2.46	6.67	2.07–21.53
Primary mental disorder diagnosis				
Bipolar disorder	1	Reference	1	Reference
Schizophrenia	3.39	1.76–6.53	4.23	1.61–11.16
Developmental disorder	1.39	0.54–3.54	1.17	0.42–3.31
Disruptive disorder	0.65	0.24	1.02	0.32–3.24
Depression	0.38	0.16–0.89	0.3	0.12–0.76
Anxiety disorder	0.68	0.30–1.51	0.61	0.20–1.82
Learning disorder	1.48	0.35–6.27	0.92	0.16–5.38
ADHD	1.64	0.85–3.20	2.65	1.07–6.60
Other mental disorder	1.33	0.51–3.48	1.26	0.42–3.72
No mental disorder diagnosis	0.78	0.24–2.57	2.2	0.75–6.48

* The multivariate logistic regression model adjusted for geographic region, MSA, median household income, and % of adults with a Bachelor’s degree or higher based on patient zip codes, in addition to the variables above.

## Appendix A

**Table A1:** Sensitivity analysis of national estimated visit rates of AP monotherapy and AP polypharmacy stratified by patient and provider characteristics between 2007 and 2011.

Characteristics	Monotherapy		Polypharmacy		*p*-Value *
	Weighted Count (in Thousands)	Weighted %	Weighted Count (in Thousands)	Weighted %	
Age					0.004
6–12 (Elementary school age)	4251	27.87	135	15.44	
13–18 Years (Adolescent)	5980	39.2	227	25.91	
19–24 (Young adult)	5023	32.93	514	58.66	
Sex					0.084
Female	5803	38.04	218	24.84	
Male	9452	61.96	659	75.16	
Race					0.365
White	12,255	80.34	760	86.62	
Black	2357	15.45	81	9.18	
Other/Unspecified	642	4.21	37	4.2	
Geographic region					0.685
Northeast	3485	22.85	273	31.1	
Midwest	3004	19.69	168	19.13	
South	5863	38.44	311	35.5	
West	2902	19.03	125	14.27	
MSA or non-MSA area					0.291
MSA	13,378	87.7	807	92.04	
non-MSA	1877	12.3	70	7.96	
Payer					0.152
Private	5666	37.45	276	31.53	
Medicaid	6576	43.46	316	36.03	
Other	2888	19.09	285	32.45	
Psychiatrist					0.317
Yes	8261	54.16	552	62.93	
No	6693	45.84	325	37.07	
Psychotherapy					0.077
Yes	4706	30.85	396	45.2	
No	10,549	69.15	481	54.8	
Other mental health counseling					0.872
Yes	3504	22.97	210	23.99	
No	11,751	77.03	667	76.01	
Mental health provider					0.002
Yes	1230	8.06	180	20.57	
No	14,025	91.94	697	79.43	
Number of non-AP (mean, SD)	2.02	0.08	2.45	0.20	0.03
Median household Income based on patient zip code					0.341
Quartile 1	3302	23.64	225	27.44	
Quartile 2	3294	23.58	78	9.57	
Quartile 3	3100	22.20	184	22.51	
Quartile 4	4271	30.58	331	40.48	
% Adults with Bachelor’s degree or higher based on patient zip code					0.894
Quartile 1	2973	21.29	154	18.78	
Quartile 2	3490	24.99	165	20.16	
Quartile 3	3103	22.22	217	26.51	
Quartile 4	4401	31.51	283	34.55	

* Chi-squared tests were used for all variables, except the number of non-AP prescriptions (*t*-test).

**Table A2:** Sensitivity analysis of univariate and multivariate logistic regressions examining factors associated with AP polypharmacy.

Characteristics	Unadjusted Odds Ratio	95% Confidence Interval	Adjusted Odds Ratio *	95% Confidence Interval
Age				
6–12 (Elementary school age)	1	Reference	1	Reference
13–18 (Adolescent)	0.54	0.28–1.05	1.50	0.50–4.50
19–24 (Young adult)	2.89	1.67–5.01	3.57	1.08–11.78
Sex				
Male	1	Reference	1	Reference
Female	0.54	0.26–1.10	0.53	0.22–1.27
Race				
White	1	Reference	1	Reference
Black	0.55	0.27–1.15	0.23	0.09–0.60
Other/Unspecified	1	0.26–3.82	1.07	0.23–5.11
Payer				
Private	1	Reference	1	Reference
Medicaid	0.82	0.41–1.62	1.45	0.62–3.37
Other	2.06	0.98–4.32	2.11	0.91–4.90
Psychotherapy	1.85	0.93–3.69	1.71	0.74–3.94
Other mental health counseling	1.06	0.53–2.11	1.02	0.47–2.25
Mental health provider	2.95	1.45–6.00	2.16	0.83–5.62
Psychiatrist	1.44	0.71–2.93	1.40	0.66–2.96
Number of non-AP				
None	1	Reference	1	Reference
One	0.91	0.49–1.69	5.28	1.53–18.20
Two	1.42	0.57–3.56	7.28	1.78–29.66
Three or More	1.25	0.63–2.46	6.30	1.94–20.47
Primary mental disorder diagnosis				
Bipolar disorder	1	Reference	1	Reference
Schizophrenia	3.39	1.76–6.53	4.53	1.69–12.13
Developmental disorder	1.39	0.54–3.54	1.21	0.43–3.40
Disruptive disorder	0.65	0.24	1.51	0.33–4.04
Depression	0.38	0.16–0.89	0.33	0.13–0.83
Anxiety disorder	0.68	0.30–1.51	0.67	0.22–2.07
Learning disorder	1.48	0.35–6.27	0.99	0.18–5.61
ADHD	1.64	0.85–3.20	2.83	1.13–7.11
Other mental disorder	1.33	0.51–3.48	1.11	0.35–3.51
No mental disorder diagnosis	0.78	0.24–2.57	2.16	0.70–6.66

* The multivariate logistic regression model adjusted for geographic region, MSA, median household income and % of adults with a Bachelor’s degree or higher based on patient zip codes, in addition to variables above.
